# The Swinging Pendulum in Treatment for Hypothyroidism: From (and Toward?) Combination Therapy

**DOI:** 10.3389/fendo.2019.00446

**Published:** 2019-07-09

**Authors:** Elizabeth A. McAninch, Antonio C. Bianco

**Affiliations:** ^1^Division of Endocrinology and Metabolism, Rush University Medical Center, Chicago, IL, United States; ^2^Section of Endocrinology and Metabolism, University of Chicago, Chicago, IL, United States

**Keywords:** thyroid, levothyroxine, liothyronine, desiccated, history

## Abstract

Thyroid hormone replacement for hypothyroidism can be achieved via several approaches utilizing different preparations of thyroid hormones, T3, and/or T4. “Combination therapy” involves administration of both T3 and T4, and was technically the first treatment for hypothyroidism. It was lauded as a cure for the morbidity and mortality associated with myxedema, the most severe presentation of overt hypothyroidism. In the late nineteenth and the early Twentieth centuries, combination therapy *per se* could consist of thyroid gland transplant, or more commonly, consumption of desiccated animal thyroid, thyroid extract, or thyroglobulin. Combination therapy remained the mainstay of therapy for decades despite development of synthetic formulations of T4 and T3, because it was efficacious and cost effective. However, concerns emerged about the consistency and potency of desiccated thyroid hormone after cases were reported detailing either continued hypothyroidism or iatrogenic thyrotoxicosis. Development of the TSH radioimmunoassay and discovery of conversion of T4-to-T3 in humans led to a major transition in clinical practices away from combination therapy, to adoption of levothyroxine “monotherapy” as the standard of care. Levothyroxine monotherapy has a favorable safety profile and can effectively normalize the serum TSH, the most sensitive marker of hypothyroidism. Whether levothyroxine monotherapy restores thyroid hormone signaling within all tissues remains controversial. Evidence of persistent signs and symptoms of hypothyroidism during levothyroxine monotherapy at doses that normalize serum TSH is mounting. Hence, in the last decade there has been acknowledgment by all thyroid professional societies that there may be a role for the use of combination therapy; this represents a significant shift in the clinical practice guidelines. Further bolstering this trend are the recent findings that the Thr92AlaD2 polymorphism may reduce thyroid hormone signaling, resulting in localized and systemic hypothyroidism. This strengthens the hypothesis that treatment options could be personalized, taking into consideration genotypes and comorbidities. The development of long-acting formulations of liothyronine and continued advancements in development of thyroid regenerative therapy, may propel the field closer to adoption of a physiologic thyroid hormone replacement regimen with combination therapy.

## Introduction

Hypothyroidism is a prevalent condition, diagnosed in most cases by an elevation in serum TSH (1). While severe myxedema has been clinically recognized since the nineteenth century, the diagnosis of lower-grade hypothyroidism has not always been straightforward (2); early diagnostic attempts relied on parameters such as a slow basal metabolic rate (BMR), low serum protein-bound iodine (PBI), or even clinical responsiveness to thyroid preparations (3). In patients for whom the diagnosis has been secured, thyroid hormone replacement has been the mainstay of therapy for over a century (1, 3). Natural thyroid preparations, i.e., thyroid extract, desiccated thyroid, or thyroglobulin, were the first pharmacologic treatments while synthetic agents were introduced later and are the standard of care today (3). Despite major progress, there remains debate as to whether a universal approach is applicable to all patients and which agent constitutes the best thyroid hormone replacement.

Combination therapy via natural thyroid preparations remained the dominant therapeutic option for the better part of the twentieth century; dosages were adjusted to resolve symptoms and to normalize BMR/PBI (4–6). Yet with this regimen, thyrotoxic side effects were not uncommon (7). In the 1970's, the clinical approach to the hypothyroid patient changed markedly based on (i) the development of immunological assays to measure serum TSH as a more reliable biochemical index of thyroid activity (8), (ii) the accessible pricing of synthetic thyroid hormone formulations, and (iii) the discovery that in humans most circulating T3 is derived via extrathyroidal conversion of T4 (3, 9). These three factors led to a dramatic change in how hypothyroidism was diagnosed and treated, such that in the last 40 years (i) measurement of serum TSH has become the cornerstone of diagnosis and therapeutic monitoring, (ii) the replacement dosage of thyroid hormone has been substantially decreased, and (iii) “monotherapy” with levothyroxine (LT4) has become a universally accepted first-line approach given its excellent safety index. LT4 monotherapy establishes normalization of serum TSH levels and symptomatic remission for a majority of patients. Of course, the foundation for the success of this regimen is largely attributed to the physiologic action of the deiodinases (10); it is widely accepted that LT4 restores the pool of prohormone, T4, and the deiodinases regulate peripheral T3 production (11).

The efficacy of LT4 monotherapy has come into question as with this approach, 10–15% of patients express dissatisfaction due to residual symptoms of hypothyroidism (12, 13), and specifically cognitive impairment (14, 15). This might not have happened in the previous era given the much higher replacement doses of thyroid hormone used prior to the institution of the serum TSH radioimmunoassay (RIA) (3). In fact, when the dose of LT4 is adjusted to maintain a normal serum TSH, the ability of the deiodinases to appropriately regulate T3 availability has been challenged by the observation that about 15% of patients receiving LT4 alone fail to achieve normal serum T3 levels (15–17). The study of a number of animal models indicate that maintaining normal serum T3 levels is a biological priority (18). Although the clinical significance of relatively low serum T3 is not well-defined (1), there is evidence demonstrating that elevating serum T3 utilizing combination therapy can have improved symptomatology for some patients (19–22). Thus, given the high prevalence of hypothyroidism and the significant proportion of patients that remain symptomatic, this represents a target for improvement of the public health. The most recent treatment guidelines have been revised to acknowledge these gaps in the approach to hypothyroid patients (Table 1) (1, 26, 27).

**Table 1:** Trends in guidelines from professional societies.

**Authors (year)**	**Professional Society**	**Rec #**	**Standard of care**	**Notes about combination therapy**	**Goal**
Singer et al. (23)	ATA		LT4 “is the treatment of choice for the routine management of hypothyroidism”	Chronic LT3 “not recommended” DT “not necessarily contraindicated”	Normalization of serum TSH
Baskin et al. (24)	AACE/ATA		“all physicians will treat clinical hypothyroidism with” LT4	“desiccated thyroid hormone, combinations of thyroid hormones, or triiodothyronine should not be used as replacement therapy” “insufficient evidence is available to know which patients with hypothyroidism, if any, would be better treated with a combination of T4 plus T3 rather than with T4 alone”	Normalization of serum TSH
Garber et al. (25)	AACE/ATA	22.1	“Patients with hypothyroidism should be treated with L-thyroxine monotherapy”	REC 22.2: “The evidence does not support using L-thyroxine and L-triiodothyronine combinations to treat hypothyroidism.” REC 22.4: “There is no evidence to support using desiccated thyroid hormone in preference to L-thyroxine monotherapy in the treatment of hypothyroidism and therefore desiccated thyroid hormone should not be used for the treatment of hypothyroidism.”	Normalization of serum TSH
Wiersinga et al. (26)	ETA	1, 2	Acknowledgment that some LT4-treated patients with normal serum TSH may have persistent symptoms	Rec 7: “L-T4 + L-T3 combination therapy might be considered as an experimental approach in compliant L-T4-treated hypothyroid patients who have persistent complaints despite serum TSH values within the reference range”	“The goal of…combination therapy is to resolve persistent complaints despite a normal TSH in L-T4-treated hypothyroid patients. In an attempt to realize this goal, it is assumed that…a euthyroid state simultaneously in all tissues of hypothyroid patients is present if serum TSH, free T4, free T3, and free T4:free T3 ratio are all within the reference range.”
Jonklaas et al. (1)	ATA	1a	“Levothyroxine is recommended as the preparation of choice for the treatment of hypothyroidism”	Rec 13c: “For patients with primary hypothyroidism who feel unwell on levothyroxine therapy alone…there is currently insufficient evidence to support the routine use of a trial of a combination of levothyroxine and liothyronine therapy…due to uncertainty in long-term risk benefit ratio of the treatment and uncertainty as to the optimal definition of a successful trial to guide clinical decision-making.”	Rec 1b: LT4 treatment goals include “(i) to provide resolution of the patients' symptoms and hypothyroid signs, including biological and physiologic markers of hypothyroidism, (ii) to achieve normalization of serum thyrotropin with improvement in thyroid hormone concentrations”
Okosieme et al. (27)	BTF	5	“L-T4 remains the treatment of choice in hypothyroidism with the aim of therapy being to restore physical and psychological well-being while maintaining normal laboratory reference range serum TSH levels”	Rec 10: “L-T4/L-T3 combination therapy in patients with hypothyroidism should not be used routinely” Rec 12: “If a decision is made to embark on a trial of L-T4/L-T3 combination therapy in patients who have unambiguously not benefited from L-T4, then this should be reached following an open and balanced discussion of the uncertain benefits, likely risks of over-replacement and lack of long-term safety data.”	Normalization of serum TSH

*AACE, American Academy of Clinical Endocrinologists; ATA, American Thyroid Association; BTF, British Thyroid Association; ETA, European Thyroid Association; LT4/L-T4, levothyroxine; LT3/L-T3, liothyronine; DT, desiccated thyroid; TSH, thyroid-stimulating hormone*.

There are new insights into the molecular mechanisms underlying the relatively lower serum T3 associated with LT4 monotherapy (28), namely that the hypothalamus exhibits altered D2 ubiquitination, explaining the inability of LT4 alone to normalize serum T3 levels (29). Only steady delivery of LT4 and LT3 in thyroidectomized rats fully normalizes serum and tissue T3 levels (30), as well as T3-dependent metabolic markers and gene expression profiles in this animal model (29). In humans, a large systematic review and meta-analysis recently showed that T3-dependent metabolic markers, such as total and LDL cholesterol, remain significantly higher in LT4-treated hypothyroid patients with normal serum TSH levels compared to healthy controls (31). A prevalent genetic polymorphism in the type 2 deiodinase, Thr92AlaD2, disrupts cellular morphology, has a prolonged half-life, is associated with ER stress and may exhibit decreased catalytic activity (32–34). Although further studies are needed to confirm these mechanisms and the clinical implications of a relatively low serum T3 need to be further defined, the available clinical evidence suggest that LT4 monotherapy may not represent a universal “replacement” for endogenous euthyroidism.

## Need for Thyroid Replacement Established, Treatment Strategies Refined

Cases describing the clinical syndromes resulting from severe hypothyroidism, namely cretinism in children and myxedema in adults, were reported in the mid-nineteenth century (35–38) but were not initially connected with a deficiency from the thyroid gland (35, 39). The causal relationship was not understood until surgeons noted incident myxedema following total thyroidectomy (40, 41); milder symptoms consisting of a “*dull, listless, mental state*” were noted when only partial thyroidectomy was performed (42). By the late 1890's, its clinical features were well-described and its epidemiology better understood; myxedema could be sporadic, of insidious onset, occurred more commonly in women, and its prevalence variable by region (43).

Initial treatment strategies for hypothyroidism were largely insufficient, basically supportive and symptom-directed therapies: “*protection against cold, persistent use of hot baths with vigorous friction did much good;…The more favorable surroundings in hospital conferred temporary benefit on some cases, and removal to a mild and genial climate on others*” (44). The significant morbidity and mortality in the absence of efficacious treatment was clear, “*the progress of the disease is not readily affected by any remedy. The prognosis is altogether unfavorable*,” (45) and thus the need to “replace” the thyroid was established. Thyroid transplant (46–50), seemingly the most divergent from contemporary approaches, had some early successes as many patients had improvement after receiving animal (sheep or goat, preferably pregnant) or human thyroid glands taken from patients with Graves' disease or goiter. Grafts were typically transplanted into the tibia or the abdominal cavity. For many patients, symptoms recurred and the procedure was repeated in some up to four times (51). Due to the rapid and transient improvement observed, “*too soon, therefore for the gland to have become vascularized and functionally active in its new situation*” (44), it was hypothesized that symptoms improved by absorption of the secretions of the donor gland (47, 52). Whereas, thyroid transplant was likely to provide uncertain quantities of the two hormones, T4 and T3, it could nevertheless be considered to be the earliest examples of a form of “combination therapy.”

Trials of the first pharmacologic strategies included other combination therapies: intravenous/subcutaneous administration of thyroid extract was utilized by Murray to treat myxedema (44, 53–55), per oral thyroid extract (56, 57), or the consumption of raw or cooked thyroid gland (55, 58, 59). These strategies saw remarkable successes, “*the results are perfectly marvelous*” (60). Oral thyroid replacement strategies won favor as their successes were undeniable and without the morbidities and relapse rates associated with transplant. However, it was noted early on that there could be side effects of treatment: “*thyroid gland…is responsible for distressing and even alarming symptoms*,” but the details were not fully described (61). Progress toward a modern thyroid transplant treatment modality is ongoing given that functional thyroid tissue can be generated from stem cells by over-expression of the thyroid transcription factors (62–64), in which case the field would have come full circle (65).

Thyroxine was crystallized in 1915 by Kendall (66), its chemical structure identified (67), and was administered successfully as an IV therapy by 1925 (68). This provided the basis for the development of synthetic LT4 (69, 70), which was shown to be efficacious in the treatment of myxedema (71) and in patients who failed to respond to desiccated thyroid treatment where clinical response was defined as BMR and restoration of ovulation/fertility (72). In 1952, serum T3 was discovered by Gross and Pitt-Rivers (73, 74). Serum PBI emerged as a diagnostic test and therapeutic marker, reflecting the combined amounts of circulating, protein-bound, T4 and T3. In the era prior to the availability of the TSH assay, this was the most specific diagnostic tool (75). However, PBI was limited in terms of monitoring a response to treatment as the “*concentration of the PBI associated with restoration of a normal metabolic state depends upon the particular thyroid hormone employed*” (76). For example, LT3 was reported as correcting BMR without much increase in PBI (77), whereas LT4 increased PBI sometimes to above the upper limit of the normal range (78), and combination LT4 + LT3 and desiccated thyroid had the advantage of normalizing PBI (79).

As such, mixtures of LT4 and LT3 administered concomitantly were proposed and developed, “*the ideal thyroid hormone preparation should combine* [LT4 and LT3] *in physiologic proportions to simulate the metabolic effects of endogenous thyroid hormone secretions*” (80). These investigators concluded that a mixture of 175 mcg LT4:50mcg LT3 was ideal because it optimized both BMR and PBI (80), but other investigators proposed ratios on the order of about 9:1 (81). Thus, despite the development of LT4, combination therapy via LT4 + LT3 or desiccated thyroid was still the preferred regimen (3).

Clinical trials were designed to assess efficacy and dose equivalency between the multiple forms of thyroid hormone replacement. Importantly, (i) these were not designed as superiority trials, (ii) outcomes assessed included normalization of PBI and/or BMR, and (iii) doses were dramatically higher than used today (3). Therefore, it is difficult to determine whether any thyrotoxic side effects were related to the type of the agent used or a consequence of its high dosage. For example, in studies utilizing doses of LT3 75–100 mcg/day, angina and congestive heart failure were observed (82); in another trial, palpitations, irritability, nervousness, dizziness, tremor, and perspiration were observed on LT4 (80 mcg) plus LT3 (20 mcg) daily (83).

**Figure 1:**
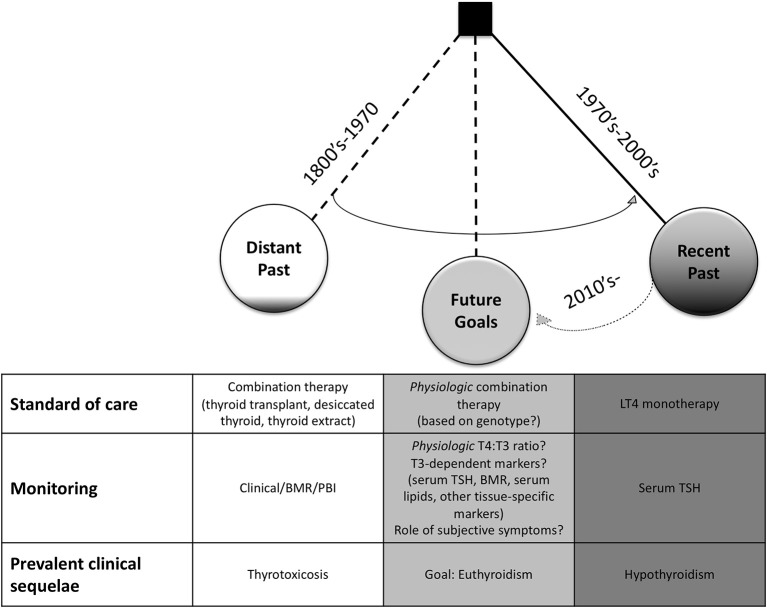
Historical Trends in the Treatment of Hypothyroidism. In the late 1800's–mid 1900's, combination therapy via thyroid transplant, thyroid feeding, thyroid extracts, thyroglobulin, or desiccated thyroid was preferred. Treatment was monitored by clinical response, basal metabolic rate (BMR), and/or serum protein-bound iodine (PBI). Thyrotoxic symptoms were prevalent in early clinical trials. In juxtaposition, levothyroxine (LT4) monotherapy to achieve normal serum TSH levels was adopted as the standard of care in the 1970's. It has become more recognized that patients on this regimen can have residual signs and symptoms of hypothyroidism. Thus, underscoring the need for new, physiologic thyroid replacement regimens with the goal to restore thyroid hormone signaling within all tissues. With the recognition that genetic polymorphisms may play a significant clinical role, personalized medicine will likely be integrated into future clinical trials and treatment regimens.

Despite these concerns, it was noted that these thyrotoxic side effects were typically remediable by simple reduction in dosage (82, 84), so combination therapy, usually by desiccated thyroid, remained the preparation of choice (85) through the mid-1970's for the treatment of hypothyroidism (3). This preference was reinforced by the unique ability of desiccated thyroid to reproduce a normal PBI as compared with LT3 or LT4 monotherapies, making biochemical monitoring more straightforward (Figure 1) (7, 80). In 1965, approximately four out of every five prescriptions for thyroid hormone were for natural preparations in the US (86).

Prominent manufacturers of natural thyroid products including both desiccated thyroid (87) and thyroglobulin (88), boasted about their “*double standardization*” (87) methods to ensure “*unvarying metabolic activity*” (88) between batches; this included (i) chemical assessment of iodine content to adhere to the standards of the British or United States Pharmacopeia (BP or USP) as well as (ii) biologic activity assessed by change in oxygen consumption in treated guinea pigs (89) or its ability to reduce the size of animal goiters (90). Despite these efforts, clinicians remained rightfully concerned regarding inconsistencies in the potency of these tablets (91). Even as early as 1911, physicians understood that there was variability within natural thyroid preparations, “*there are probably some [preparations of thyroid] on the market that are inactive. It is only natural that the properties and activity of the gland should vary in different animals, according to their age and sex, and probably even according to their pasturage*” (92). Despite adherence to iodine content standards, some batches had varying potency (76), such that tablets contained nearly double potency, and others had almost no detectable metabolic activity (93). Also, humidity limited the shelf-life of desiccated tablets (84). There were reports of patients failing to respond to desiccated thyroid altogether as their tablets contained no active thyroid hormone (94–96). This led to claims that desiccated thyroid was dangerous and “*that its manufacture be abolished*” (97); it became viewed by many as “*obsolete*” as it “*possesses no uniquely desirable properties and should, therefore be retired to the place that it has earned in medical history*” (98). It was not until 1985 that the revision of the USP standard from iodine content to T3/T4 content established stable potency (86).

## Transition Away From Combination Therapy

Despite growing discontent with variable potency of natural thyroid products (93), as well as lowering in cost of LT4 such that the two treatments were approximately equivalent (99), physicians hesitated to use LT4 monotherapy, concerned that it could result in a relative T3 deficiency (84, 91). However, the landmark discovery of peripheral T4-to-T3 conversion in athyreotic humans by Braverman et al. obviated this concern (9) and provided the foundation for the hypothesis that LT4 could replace prohormone pool and the deiodinases would regulate availability of active T3 (11). This discovery had a major influence on the prescribing practices of physicians such that within about a decade there was a major transition toward LT4 as the first-line therapy in hypothyroidism (3, 86, 90).

The TSH RIA was developed by Utiger almost simultaneously (8). Clinicians were able to titrate therapy to achieve a serum TSH within the normal range as a specific marker of thyroid hormone replacement adequacy (100–102). This came with the caveat that early TSH assays were not able to distinguish between normal and low serum TSH levels (103). Thus, patients treated to a suppressed TSH (normal for that method) could only be differentiated through employment of a second test, the thyrotropin-releasing hormone (TRH) stimulation test, which could identify over-treated patients (suppressed TSH) by their subnormal response to exogenous TRH (104). For patients that were once treated with doses that normalized their symptoms, BMR, or PBI, the utilization of serum TSH (associated with the TRH stimulation test) revealed such doses to be typically supratherapeutic (14, 103, 105, 106). Whereas, prior to the institution of the TSH assay, typical maintenance doses of LT4 were in the 200–500 mcg/day range, doses were now typically closer to 100–150 mcg/day (3, 103, 105, 106).

Soon thereafter was the development of RIAs for measurement of serum T3 (107, 108) and T4 (109). With the availability of these assays, it was observed that LT4 could normalize both T4 and T3 levels at the expense of a high T4/T3 ratio. LT3, desiccated thyroid, thyroglobulin, and LT4 + LT3 combination all typically resulted in low or low-normal T4 values with usually elevated T3 levels (90). In particular, it was noted that desiccated thyroid resulted in a T3 peak occurring about 2–5 h after administration that corresponds to thyrotoxic symptoms in some patients (99). That a single daily dose of oral LT4 resulted in nearly seemingly physiologic, stable blood levels of T4 and T3 throughout the day (107) was understood to be a result of a steady rate of conversion of T4-to-T3 (110, 111). In less than a decade after discovery of peripheral T4-to-T3 conversion and with implementation of RIAs to specifically quantitate serum TSH, T4, and T3, normalization of TSH with LT4 became the new standard of care (Figure 1) (3, 112). These findings left many clinicians advocating not only for LT4 to be the first-line therapy, but that patients previously treated with desiccated thyroid be transitioned to LT4 (99).

## Defining “Euthyroidism” and Revisiting Combination Therapy

Following the transition to LT4 monotherapy and reduction in replacement dose to achieve a normal TSH, clinicians noted several important differences in the ability of this regimen to normalize markers of hypothyroidism such as BMR, serum cholesterol, and patient satisfaction (3). In many LT4-treated hypothyroid patients with a normal TSH, the BMR remained at about −10% of that of normal controls (113). Whereas, LT4 treatment at doses that normalize BMR, can suppress the serum TSH (90, 103, 105, 106, 114). Recent investigations have confirmed that energy expenditure is only normalized in LT4 treatment at doses that suppress the serum TSH (115). Another study found that energy expenditure does not differ between groups treated with LT4 doses to result in either high-normal or low-normal serum TSH levels (116). Hypothyroidism is a secondary cause of dyslipidemia, typically manifesting in elevation of LDL and total cholesterol levels (31), however, it was noted that normalization of LDL in LT4-treated hypothyroid patients can require TSH-suppressive doses (117, 118). Complaints from dissatisfied patients treated with LT4 monotherapy at doses to normalize the serum TSH were often dismissed as unrelated to their thyroid condition (119), or attributed to non-compliance (120), as symptoms are non-specific and can overlap with other common conditions including menopause, depression, and chronic fatigue syndrome (2). However, LT4-treated patients display significant impairment in psychological well-being compared to controls of similar age and sex (14). To assess whether this was a result of trends toward lower doses of LT4, measures of well-being were tracked on various doses and it was found that the highest well-being is achieved at doses resulting in a suppressed TSH (121). However, such findings were not always reproducible (122). Indeed, it has been shown in a large population study that LT4-treated patients exhibit higher BMIs and take more statins and anti-depressants than TSH-matched, healthy controls (15); this association could have been impacted by confounding and thus further investigation is indicated to confirm these results (15). Thus, in LT4 monotherapy, defining euthyroidism as normal serum TSH has flaws as other clinical parameters may not be normalized.

Overtreatment associated with a low serum TSH, is associated with increased cardiovascular and skeletal risks (1), thus in the current guidelines a goal of therapy remains achievement of normal serum TSH levels (Table 1) (1, 26, 27). A small study found that patients perceived that their physicians were overly reliant on serum TSH levels and that this was a barrier to them receiving optimal care (123). Prescribing patterns have changed such that serum TSH level at time of initial treatment has been decreasing (124, 125) yet this may not improve quality of life or thyroid-related symptoms (126). Thus, reconciliation between optimization of patient outcomes without the increased risks of overtreatment remains a unique challenge in the field.

It should be noted that assessment and interpretation of serum T3 levels presents significant limitations as well due to (i) the difficulties accurately measuring serum free T3 with standard clinical lab assays (18), (ii) the fact that serum T3 levels may not fully represent intracellular T3 due to intracellular deiodination (10), and (iii) other non-thyroidal illnesses are known to result in low serum T3 (1, 18). In a study of 42 patients, assessment of serum T3 at baseline and during combination therapy did not predict positive, symptomatic response (127). Thus, the clinical utility of serum T3 measurements is unknown (1).

In a clinical trial of combination therapy with LT4 + LT3 to establish normal serum TSHs, there was improvement in psychological parameters (19). In another study comparing LT4 monotherapy to desiccated thyroid, in which both groups had a normal TSH, 48% of patients preferred desiccated thyroid over LT4 monotherapy (18.6% preferred LT4) and those patients preferring dessicated thyroid also experienced about 4 pound weight loss over the 16 week treatment period (21). Indeed, many clinical trials show subjective “preference” for combination therapy without positive objective results when utilizing quality of life and/or thyroid-specific questionnaires (19, 21, 128, 129). This suggests that these questionnaires may not be capturing the parameters improved by combination therapy, and opens yet another path for further research. Benefits with combination therapy have not been reproduced in all populations, and many studies fail to demonstrate superiority of combination therapy (1, 14). This may be related to the pharmacologic properties of available oral LT3 preparations. There are theoretical concerns about adverse events with LT3 treatment, but in one observational study over 17 years, there were no increased cardiovascular or skeletal risks (130).

Modern professional societies have synthesized their best practice guidelines for hypothyroidism. These guidelines have been evolving away from a universal approach with LT4 monotherapy (23, 24, 131) and toward an approach that accepts a therapeutic trial of combination therapy for select patients (Table 1) (1, 26, 27). In 1995, the American Thyroid Association (ATA) recommended LT4 monotherapy, and recommended against LT3 due to risk of iatrogenic thyrotoxicosis (23). In their conjoint guideline, the ATA and the American Association of Clinical Endocrinologists (AACE) stated that “*all physicians will treat clinical hypothyroidism with levothyroxine*” (24), and also recommended against combination therapy (Table 1). These recommendations were similarly upheld in the ATA/AACE guidelines in 2012 (131). The 1995, 2002, and 2012 guidelines all recommended normalization of serum TSH as the treatment goal (Table 1; Figure 1) (23, 24, 131).

However, in 2012, the European Thyroid Association (ETA) published guidelines (26) in contrast to those of ATA/AACE (131). These ETA guidelines acknowledged that some LT4-treated patients with normal serum TSHs may have persistent symptoms based on increased verbalization from patient advocacy groups and supportive evidence from some clinical trials (26, 128). These guidelines also clearly documented hypotheses for treatment dissatisfaction among hypothyroid individuals: causes related to disease chronicity, associated autoimmune diseases, thyroid autoimmunity, inadequate LT4 dosing, and inadequacy of LT4 treatment modality (26). The acknowledgment of dissatisfaction among a significant proportion of individuals and documentation of possible etiologies to stimulate future research (26), seem to be in stark contrast to the previous paradigm of universal LT4 monotherapy (Table 1) (23, 24, 131). The ETA offered a second-line approach for these symptomatic individuals: using LT4 + LT3 combination therapy for select individuals in “carefully executed” therapeutic trials. The ATA then built upon these recommendations given the new evidence that polymorphisms in the deiodinases can be associated with differences in serum thyroid hormone levels (132) and acknowledgment that some LT4-treated individuals have relatively low serum T3 concentrations (1, 16, 17, 133–135). The ATA did note that there were inconsistent findings from clinical trials of combination therapy, thus superiority of combination therapy had not been established (1). Goals of therapy included normalization of serum TSH and “*to provide resolution of the patients' symptoms and hypothyroid signs, including biological and physiologic markers of hypothyroidism*” (1). Although these markers were not well-defined, this represented a significant shift compared to prior ATA guidelines (Table 1) (23, 131). The British Thyroid Foundation likewise recommended therapeutic trial of LT4 + LT3 combination therapy to “*restore physical and psychological well-being”* (27). Despite consensus from these societies that LT4 monotherapy remain as first-line, a recent survey found that at least 58% of clinicians would prescribe a trial of combination therapy for specific clinical scenarios in which LT4-treated patients with normal serum TSH exhibited residual symptoms (136).

## New Evidence May Justify Combination Therapy

The importance of investigating the benefits associated with combination therapy in humans is highlighted by findings in an animal model of hypothyroidism. As in humans, LT4 monotherapy for athyreotic rats results in a high T4:T3 ratio at doses sufficient to normalize serum TSH levels (29). Yet, the brain, liver and skeletal muscle tissues of these LT4-treated animals exhibit markers of localized hypothyroidism (29), likely due the inability of LT4 monotherapy to restore tissue levels of T3 (30). This occurs as a direct consequence of the relatively high T4 concentration in these tissues: D2 downregulation is T4-mediated. In the hypothalamus, as a result of localized reduction in D2 ubiquitination, there is increased sensitivity to T4 levels, explaining the ability of the TSH to be normalized despite relatively lower levels of serum T3. Thus, only combination therapy with stable release LT4 + LT3 normalized all parameters of thyroid hormone homoeostasis (29) including serum and tissue T3 levels in rodents (30). In humans, LT4 monotherapy results in a high T4:T3 ratio (15, 16, 120, 137), thus underscoring the importance in establishing its clinical significance (1, 18, 31, 138). A large systematic review and meta-analysis of T3-dependent markers in hypothyroid humans treated with LT4 monotherapy, showed that LDL (3.31 ± 1.64 mg/dL) and total cholesterol (9.60 ± 3.55 mg/dL) remain higher in LT4-treated patients than healthy controls, despite normalization of serum TSH (31). The clinical significance of this difference in serum cholesterol remains to be determined, however this may justify well-designed clinical trials of combination therapy utilizing tissue-specific markers of thyroid status as outcome measures.

One factor that has been associated with response to combination therapy in multiple clinical trials is the Thr92Ala polymorphism in the type 2 deiodinase, where carriers can exhibit improved quality of life measures and preference for combination therapy (20, 22). This has led to the logical hypothesis that Thr92AlaD2 could be associated with localized and/or systemic hypothyroidism, yet results from clinical trials have not been consistently supportive (139–141). Multiple groups have demonstrated normal *in vitro* Thr92AlaD2 enzyme kinetics (142, 143), but other groups have found evidence of reduced enzymatic activity at the cellular and organism level (144). The Thr92AlaD2 protein has been found to disturb cellular physiology: it had a longer half-life, localized in the Golgi apparatus and significantly alter the transcriptome while stably expressed *in vitro* (33). In the same study, it was demonstrated that in the human temporal pole the transcriptome was similarly altered, resulting in a proposed 81-gene fingerprint of Thr92AlaD2 expression (33). An unexpected finding was that this transcriptome from human temporal pole samples shared expression patterns found in neurodegenerative diseases. Indeed, in a large cohort, African American carriers of Thr92AlaD2 exhibited about 30% greater risk of developing Alzheimer's disease (32), suggesting that the study of Thr92AlaD2 transcends the thyroid field. In a novel animal model of Thr92AlaD2 expression, there was evidence of ER stress and neurocognitive dysfunction; with administration of LT3 to animals with intact endogenous thyroids, the phenotype improved, bolstering support for the positive findings in many clinical trials (34). As the molecular basis for the Thr92AlaD2 observations is better characterized, it remains to be confirmed whether hypothyroid carriers may benefit from individualized therapies. If so, then the notion of personalized medicine, based on genotype, may define the future of management in hypothyroidism (3).

A slow-release oral form of LT3 was recently developed and applied in hypothyroid rats where it was found to provide stable, normal serum T3 levels (145). Results from human trials with this agent have yet to be determined, but this provides great hope that future high quality, randomized, controlled clinical trials will establish whether steady-dose LT3 + LT4 combination therapy is superior to LT4 monotherapy in terms of its ability to normalize all parameters of thyroid hormone homoeostasis, including tissue markers, mood, and cognition. Of course such trials would need to evaluate whether patients with the Thr92AlaD2 polymorphism, and other polymorphisms that could be relevant in thyroid hormone signaling, respond uniquely to treatments.

In conclusion, whereas combination therapy once dominated, this trend was largely abandoned in the 1970's due to evidence of iatrogenic thyrotoxicosis and concerns of consistency. In addition, a consequence of the availability of sensitive TSH assays was a dramatic reduction in thyroid hormone replacement dosage. Discovery of peripheral T4-to-T3 conversion provided initial physiologic justification for LT4 monotherapy. Clinical practice trended away from natural thyroid preparations and toward LT4 monotherapy given at doses to normalize the serum TSH. This transition was associated with the emergence of a population of patients with residual signs and symptoms of hypothyroidism and relatively lower serum T3 levels, despite normalization of serum TSH levels. New evidence that genetic polymorphisms may affect thyroid hormone signaling may substantiate objective evidence of residual localized and/or systemic hypothyroidism in a proportion of the population. The development of long acting formulations of LT3 to result in stable serum T3 levels may bolster development of a physiologic thyroid hormone replacement regimen to better mimic endogenous euthyroidism.

## Author Contributions

EM performed the literature review, drafted the manuscript, table, and figure, and edited the manuscript. AB contributed to hypothesis generation and edited the manuscript.

### Conflict of Interest Statement

AB is a consultant for Synthonics Inc, BLA Technology LLC, and Allergan LLC; he served as consultant for Sentier LLC during 2018. The remaining author declares that the research was conducted in the absence of any commercial or financial relationships that could be construed as a potential conflict of interest.

## References

[1] Jonklaas J Bianco AC Bauer AJ Burman KD Cappola AR Celi FS. Guidelines for the treatment of hypothyroidism. Thyroid. (2014) 24:1670–751. 10.1089/thy.2014.0028 25266247 PMC4267409

[2] McAninch EA Glueck JS Bianco AC. Does sex bias play a role for dissatisfied patients with hypothyroidism? J Endocr Soc. (2018) 2 970–3. 10.1210/js.2018-00169 30094410 PMC6077803

[3] McAninch EA Bianco AC. The history and future of treatment of hypothyroidism. Ann Intern Med. (2016) 164 50–6. 10.7326/M15-1799 26747302 PMC4980994

[4] Stanbury JB A Constant Ferment. Ipswich, MA: The Ipswich Press (1991).

[5] Janney NW. The diagnosis of hypothyroidism. Cal State J Med. (1921) 19:313–6. 18738529 PMC1516979

[6] Means JH Studies of the basal metabolism in disease and their importance in clinical medicine. Bos Med Surg J. (1916) 174:864–70. 10.1056/NEJM191606151742402

[7] Williams RH The thyroid. In: Williams RH editor. Textbook of Endocrinology. Philadelphia, PA: Saunders Company (1955). p. 99–220.

[8] Utiger RD. Thyrotrophin radioimmunoassay: another test of thyroid function. Ann Int Med. (1971) 74:627–9. 10.7326/0003-4819-74-4-627 5551168

[9] Braverman LE Ingbar SH Sterling K. Conversion of thyroxine (T4) to triiodothyronine (T3) in athyreotic subjects. J Clin Invest. (1970) 49:855–64. 10.1172/JCI106304 4986007 PMC535757

[10] Gereben B McAninch EA Ribeiro MO Bianco AC. Scope and limitations of iodothyronine deiodinases in hypothyroidism. Nat Rev Endocrinol. (2015) 11:642–52. 10.1038/nrendo.2015.155 26416219 PMC5003781

[11] Larsen PR Ingbar S The thyroid. In: Wilson JD Foster DW editors. Textbook of Endocrinology. Philadelphia, PA: Saunders WB Co (1992). p. 357–487.

[12] Canaris GJ Manowitz NR Mayor G Ridgway EC. The colorado thyroid disease prevalence study. Arch Intern Med. (2000) 160:526–34. 10.1001/archinte.160.4.526 10695693

[13] Peterson SJ Cappola AR Castro MR Dayan C Farwell AP Hennessey JV. An online survey of hypothyroid patients demonstrates prominent dissatisfaction. Thyroid. (2018). 28:707–21. 10.1089/thy.2017.0681 29620972 PMC6916129

[14] Saravanan P Chau WF Roberts N Vedhara K Greenwood R Dayan CM. Psychological well-being in patients on 'adequate' doses of l-thyroxine: results of a large, controlled community-based questionnaire study. Clin Endocrinol. (2002) 57:577–85. 10.1046/j.1365-2265.2002.01654.x 12390330

[15] Peterson SJ McAninch EA Bianco AC. Is a normal TSH synonymous with euthyroidism in levothyroxine monotherapy? J Clin Endocrinol Metab. (2016) 101:4964–73. 10.1210/jc.2016-2660 27700539 PMC6287526

[16] Gullo D Latina A Frasca F Le Moli R Pellegriti G Vigneri R. Levothyroxine monotherapy cannot guarantee euthyroidism in all athyreotic patients. PLoS ONE. (2011) 6:e22552. 10.1371/journal.pone.0022552 21829633 PMC3148220

[17] Ito M Miyauchi A Morita S Kudo T Nishihara E Kihara M. TSH-suppressive doses of levothyroxine are required to achieve preoperative native serum triiodothyronine levels in patients who have undergone total thyroidectomy. Euro J Endocrinol. (2012) 167:373–8. 10.1530/EJE-11-1029 22711760

[18] Abdalla SM Bianco AC. Defending plasma T3 is a biological priority. Clin Endocrinol. (2014) 81:633–41. 10.1111/cen.12538 25040645 PMC4699302

[19] Bunevicius R Kazanavicius G Zalinkevicius R Prange AJ Jr. Effects of thyroxine as compared with thyroxine plus triiodothyronine in patients with hypothyroidism. N Engl J Med. (1999) 340:424–9. 10.1056/NEJM199902113400603 9971866

[20] Panicker V Saravanan P Vaidya B Evans J Hattersley AT Frayling TM Dayan CM. Common variation in the DIO2 gene predicts baseline psychological well–being and response to combination thyroxine plus triiodothyronine therapy in hypothyroid patients. J Clin Endocrinol Metab. (2009) 94:1623–9. 10.1210/jc.2008-1301 19190113

[21] Hoang TD Olsen CH Mai VQ Clyde PW Shakir MK. Desiccated thyroid extract compared with levothyroxine in the treatment of hypothyroidism: a randomized, double-blind, crossover study. J Clin Endocrinol Metab. (2013) 98:1982–90. 10.1210/jc.2012-4107 23539727

[22] Carle A Faber J Steffensen R Laurberg P Nygaard B Hypothyroid patients encoding combined MCT10 and DIO2 gene polymorphisms may prefer L-T3 + L-T4 combination treatment - data using a blind randomized clinical study. Euro Thyroid J. (2017) 6:143–51. 10.1159/000469709 28785541 PMC5527224

[23] Singer PA Cooper DS Levy EG Ladenson PW Braverman LE Daniels G. Treatment guidelines for patients with hyperthyroidism and hypothyroidism. standards of care committee, american thyroid association. JAMA. (1995) 273:808–12. 10.1001/jama.273.10.808 7532241

[24] Baskin HJ Cobin RH Duick DS Gharib H Guttler RB Kaplan MM American association of clinical endocrinologists medical guidelines for clinical practice for the evaluation and treatment of hyperthyroidism and hypothyroidism. Endocrine Pract. (2002) 8:457–69. 10.4158/1934-2403-8.6.457 15260011

[25] Garber JR Cobin RH Gharib H Hennessey JV Klein I Mechanick JI. Clinical practice guidelines for hypothyroidism in adults: cosponsored by the American association of clinical endocrinologists and the american thyroid association. Endocrine Pract. (2012) 18:988–1028. 10.4158/EP12280.GL 23246686

[26] Wiersinga WM Duntas L Fadeyev V Nygaard B Vanderpump MP. 2012 ETA guidelines: the use of L-T4 + L-T3 in the treatment of hypothyroidism. Euro Thyroid J. (2012) 1:55–71. 10.1159/000339444 24782999 PMC3821467

[27] Okosieme O Gilbert J Abraham P Boelaert K Dayan C Gurnell M. Management of primary hypothyroidism: statement by the British thyroid association executive committee. Clin Endocrinol. (2016) 84:799–808. 10.1111/cen.12824 26010808

[28] McAninch EA Bianco AC. New insights into the variable effectiveness of levothyroxine monotherapy for hypothyroidism. Lancet Diabetes Endocrinol. (2015) 3:756–8. 10.1016/S2213-8587(15)00325-3 26362364 PMC5006060

[29] Werneck de Castro JP Fonseca TL Ueta CB McAninch EA Abdalla S Wittmann G Differences in hypothalamic type 2 deiodinase ubiquitination explain localized sensitivity to thyroxine. J Clin Invest. (2015) 125:769–81. 10.1172/JCI77588 25555216 PMC4319436

[30] Escobar-Morreale HF Rey F Obregon MJ Escobar GM. Only the combined treatment with thyroxine and triiodothyronine ensures euthyroidism in all tissues of the thyroidectomized rat. Endocrinology. (1996) 137:2490–502. 10.1210/en.137.6.2490 8641203

[31] McAninch EA Rajan KB Miller CH Bianco AC Systemic thyroid hormone status during levothyroxine therapy in hypothyroidism: a systematic review and meta-analysis. J Clin Endocrinol Metab. (2018) 103:4533–42. 10.1210/jc.2018-01361 PMC6226605 30124904

[32] McAninch EA Rajan KB Evans DA Jo S Chaker L Peeters RP. A common DIO2 polymorphism and alzheimer disease dementia in African and European Americans. J Clin Endocrinol Metab. (2018) 103:1818–26. 10.1210/jc.2017-01196 29481662 PMC6276710

[33] McAninch EA Jo S Preite NZ Farkas E Mohacsik P Fekete C. Prevalent polymorphism in thyroid hormone-activating enzyme leaves a genetic fingerprint that underlies associated clinical syndromes. J Clin Endocrinol Metab. (2015) 100:920–33. 10.1210/jc.2014-4092 25569702 PMC4333048

[34] Jo S Fonseca TL Bocco B Fernandes GW McAninch EA Bolin AP. Type 2 deiodinase polymorphism causes ER stress and hypothyroidism in the brain. J Clin Invest. (2019) 129:230–45. 10.1172/JCI123176 30352046 PMC6307951

[35] Lindholm J Laurberg P. Hypothyroidism and thyroid substitution: historical aspects. J Thyroid Res. (2011) 2011:809341. 10.4061/2011/809341 21760981 PMC3134382

[36] Sawin CT Introduction: Defining Myxoedema and Its Cause, Clinical Society of London: Report on Myxoedema 1888. Boston, MA: Science History Publications (1991). p. 1–14.

[37] Curling TB. Two cases of absence of the thyroid body, and symmetrical swellings of fat tissue at the sides of the neck, connected with defective cerebral development. Medico Chirurgical Trans. (1850) 33:303–6. 10.1177/095952875003300123 20895941 PMC2104224

[38] Fagge CH. On sporadic cretinism, occurring in England. Medico Chirurgical Trans. (1871) 54:155–70. 10.1177/095952877105400108 20896365 PMC2150485

[39] Gull WW On a cretinoid state supervenening in adult life in women. Trans Clin Soc Lond. (1873) 7:180–5.

[40] Horsley V The brown lectures on pathology. Br Med J. (1885) 1:111–5. 10.1136/bmj.1.1255.111 PMC2255603 20751136

[41] Kocher T Ueber kropf exstirpation und ihre folgen. Archiv Fur Klinische Chirurgie. (1883) 29:254–335.

[42] Chesky VE The Hospital Management of Goiter Patients, Diseases of the Thyroid Gland. St. Louis, MO: The Mosby Company (1929). p. 209–29.

[43] Bramwell B The clinical features of myxedema. Edinburgh Med J. (1893) 38:985–95. PMC5538479 29584327

[44] Lundie RA The treatment of myxoedema. Edinburgh Med J. (1893) 38:996–1005.

[45] Ord WM Myxoedema. In: Quain R editor. A Dictionary of Medicine. London: Longmans, Green, & Co (1882).

[46] Horsley V. Note on a possible means of arresting the progress of myxoedema, cachexia strumipriva, and allied diseases. Br Med J. (1890) 1:287–8. 10.1136/bmj.1.1519.287 20752942 PMC2207355

[47] Bettencourt R Serrano J Un cas de myxoedème traité par la greffe hypodermique du corps thyroïde d'un mouton. La Semaine Médicale. (1890) 10:294.

[48] Schiff M Résumé d'une nouvelle série d'expériences sur les effets de l'ablation des corps thyroîdes. Revue Médicale de la Suisse Romande. (1884) 4:425–45.

[49] Bircher Volkmann's Sammlung Klinische Vortrage 357. Leipzig: Rishard von Volkmann (1890).

[50] Eiselsberg V Ueber Tetanie im Ausschlusse an Kropfoperationen. Wchnschr III. Wien klin. (1890). p. 48.

[51] Kocher A. The treatment of hypothyroidism by thyroid transplantation. Br Med J. (1923) 2:560–1. 10.1136/bmj.2.3274.560 20771297 PMC2317016

[52] Bettencourt R Serrano J Un cas de myxoedème (cachexie pachydermique) traité par la greffe hypodermique du corps thyoïde d'un mouton. Compte Rendu de la 19me Session de l'Association Française pour l'Avancement des Sciences. Limoges Part. (1890) 2:683–90.

[53] Murray GR. Note on the treatment of myxoedema by hypodermic injections of an extract of the thyroid gland of a sheep. Br Med J. (1891) 2:796–7. 10.1136/bmj.2.1606.796 20753415 PMC2273741

[54] Murray GR. The life-history of the first case of myxoedema treated by thyroid extract. Br Med J. (1920) 1:359–60. 10.1136/bmj.1.3089.359 20769820 PMC2337775

[55] Vermeluen F The treatment of myxoedema by feeding with thyroid glands. Br Med J. (1893) 1:266 10.1136/bmj.1.1675.266 PMC2402891 20754152

[56] Ord WM White E. Clinical remarks on certain changes observed in the urine in myxoedema after the administration of glycerine extract of thyroid gland. Br Med J. (1893) 2:217. 10.1136/bmj.2.1700.217 20754379 PMC2422016

[57] Fox EL. A case of myxoedema treated by taking extract of thyroid by the mouth. Br Med J. (1892) 2:941. 10.1136/bmj.2.1661.941 20753901 PMC2421284

[58] Mackenzie HW. A case of myxoedema treated with great benefit by feeding with fresh thyroid glands. Br Med J. (1892) 2:940–1. 10.1136/bmj.2.1661.940 20753900 PMC2421275

[59] Baber EC. Feeding with fresh thyroid glands in myxoedema. Br Med J. (1893) 1:10. 10.1136/bmj.1.1671.10 20753988 PMC2404358

[60] The Chemist and Druggist Supplement (1893). 43, p. 180.

[61] Dawson R Correspondence. Montreal Med J. (1893) 22:437–9.

[62] Ma R Latif R Davies TF. Thyroid follicle formation and thyroglobulin expression in multipotent endodermal stem cells. Thyroid. (2013) 23:385–91. 10.1089/thy.2012.0644 23360087 PMC3610443

[63] Ma R Latif R Davies TF. Human embryonic stem cells form functional thyroid follicles. Thyroid. (2015) 25:455–61. 10.1089/thy.2014.0537 25585054 PMC4390159

[64] Antonica F Kasprzyk DF Opitz R Iacovino M Liao XH Dumitrescu AM. Generation of functional thyroid from embryonic stem cells. Nature. (2012) 491:66–71. 10.1038/nature11525 23051751 PMC3687105

[65] Hollenberg AN Choi J Serra M Kotton DN. Regenerative therapy for hypothyroidism: mechanisms and possibilities. Mol Cell Endocrinol. (2017) 445:35–41. 10.1016/j.mce.2016.11.012 27876515 PMC5373653

[66] Kendall EC. Landmark article, June 19, 1915. The isolation in crystalline form of the compound containing iodin, which occurs in the thyroid. its chemical nature and physiologic activity. By E.C. Kendall. JAMA. (1983) 250:2045–6. 10.1001/jama.250.15.2045 6352971

[67] Harington CR. Chemistry of thyroxine: isolation of thyroxine from the thyroid gland. Biochem J. (1926) 20:293–9. 10.1042/bj0200293 16743658 PMC1251713

[68] Boothby WM Sandiford I Sandiford K Slosse J The effect of thyroxin on the respitaory and nitrogenous metabolism of normal and myxedematous subjects. Tr A Am Phys. (1925) 195:725–56. 10.1007/BF01958332

[69] Chalmers JR Dickson GT Elks J Hems BA The synthesis of thyroxine and related substances. Part V. A synthesis of L-thyroxine from L-tyrosine. J Chem Soc. (1949) 3424–38. 10.1039/jr9490003424

[70] Borrows ET Clayton JC Hems BA The synthesis of thyroxine and related substances. Part I. the preparation of tyrosine and some of its derivatives, a new route to thyroxine. J Chem Soc. (1949) 1949:S185–90. 10.1039/jr949000s185

[71] Hart FD Maclagan NF. Synthetic thyroxine in the treatment of myxoedema. J Endocrinol. (1950) 6:xxxiv. 14774506

[72] Abarbanel AR. Thyroid extract versus thyroxine. Critical evaluation with particular reference to functional infertility. In: Meeting of the Association for the Study of Internal Secretions: Abstracts of Papers for the Thirty-Second Meeting. Springfield, IL (1950). p. 837. 15436632

[73] Gross J Pitt-Rivers R. The identification of 3:5:3'-L-triiodothyronine in human plasma. Lancet. (1952) 1:439–41. 10.1016/S0140-6736(52)91952-1 14898765

[74] Gross J Pitt-Rivers R. Physiological activity of 3:5:3'-L-triiodothyronine. Lancet. (1952) 1:593–4. 10.1016/S0140-6736(52)90104-9 14909477

[75] Williams RH. Relation of obesity to the function of the thyroid gland, especially as indicated by the protein-bound iodine concentration in the plasma. J Clin Endocrinol Metab. (1948) 8:257–61. 10.1210/jcem-8-3-257 18912537

[76] Braverman LE Ingbar SH. Anomalous effects of certain preparations of desiccated thyroid on serum protein-bound iodine. N Engl J Med. (1964) 270:439–42. 10.1056/NEJM196402272700903 14163222

[77] Selenkow HA Asper SP Jr. The effectiveness of triiodothyronine or thyroxine administered orally in the treatment of myxedema. J Clin Endocrinol Metab. (1955) 15:285–96. 10.1210/jcem-15-3-285 14353993

[78] Sturnick MI Lesses MF A comparison of the effect of desiccated thyroid and sodium levothyroxine on the serum protein-bound iodine. N Engl J Med. (1961) 264:608–9. 10.1056/NEJM196103232641207

[79] Ingbar SH Woeber KA Thyroid hormone deficiency. In: Williams RH editor. Textbook of Endocrinology. Philadelphia, PA: Saunders Company (1968). p. 232–58.

[80] Wool MS Selenkow HA. Physiologic combinations of synthetic thyroid hormones in myxedema. Clin Pharmacol Ther. (1965) 6:710–5. 10.1002/cpt196566710 4158675

[81] Taylor S A new thyroid preparation. Lancet. (1961) 277:341 10.1016/S0140-6736(61)91516-1

[82] Frawley TF McClintock JC Beebe RT Marthy GL. Metabolic and therapeutic effects of triiodothyronine. J Am Med Assoc. (1956) 160:646–52. 10.1001/jama.1956.02960430036007 13286110

[83] Smith RN Taylor SA Massey JC. Controlled clinical trial of combined triiodothyronine and thyroxine in the treatment of hypothyroidism. Br Med J. (1970) 4:145–8. 10.1136/bmj.4.5728.145 4097650 PMC1819870

[84] Werner SC Treatment; myxedema coma; nonspecific uses of thyroid medication. In: Werner SC Ingbar SH editors. The Thyroid: A Fundamental and Clinical Text. New York, NY: Harper & Row (1971). p. 832–8.

[85] Werner SC. Pharmacology; treatment. In: Werner SC editor. The Thyroid: A Fundamental and Clinical Tex. New York, NY: Harper & Row (1962). p. 817–25.

[86] Kaufman SC Gross TP Kennedy DL. Thyroid hormone use: trends in the United States from 1960 through 1988. Thyroid. (1991) 1:285–91. 10.1089/thy.1991.1.285 1841728

[87] Laboratories TA The Thyroid Gland and Clinical Application of Medicinal Thyroid, USA. Chicago, IL: Armour Laboratories (1943).

[88] Laboratories W-C Thyroid in Therapeutics. Morris Plains, NJ: Warner-Chilcott (1963).

[89] Billewicz WZ Chapman RS Crooks J Day ME Gossage J Wayne E Young JA. Statistical methods applied to the diagnosis of hypothyroidism. Quart J Med. (1969) 38:255–66. 4181088

[90] Cobb WE Jackson IM. Drug therapy reviews: management of hypothyroidism. Am J Hosp Pharm. (1978) 35:51–8. 10.1093/ajhp/35.1.51 341699

[91] Lavietes PH Epstein FH. Thyroid therapy of myxedema: a comparison of various agents with a note on the composition of thyroid secretion in man. Ann Intern Med. (1964) 60:79–87. 10.7326/0003-4819-60-1-79 14104859

[92] Waller HE General Considerations, Theory and Practice of Thyroid Therapy: Being Some Experiences of the Results of Thyroid Medication, With Deductions Concerning the Influence of Thyroid Secreiton in Health and Disesase, and Certain Effects of Drugs and Various Circumstances Upon Thyroid Secretion: A Book for General Practitioners. New York, NY: William Wood and Company (1911). p. 141–54.

[93] Mangieri CN Lund MH. Potency of United States pharmacopeia dessicated thyroid tablets as determined by the antigoitrogenic assay in rats. J Clin Endocrinol Metab. (1970) 30:102–4. 10.1210/jcem-30-1-102 5409525

[94] Macgregor AG. Why does anybody use thyroid B.P.? Lancet. (1961) 1:329–32. 10.1016/S0140-6736(61)91498-2 13764789

[95] Catz B Ginsburg E Salenger S. Clinically inactive thyroid U.S.P. a preliminary report. N Engl J Med. (1962) 266:136–7. 10.1056/NEJM196201182660308 13877407

[96] Rees-Jones RW Rolla AR Larsen PR. Hormonal content of thyroid replacement preparations. J Am Med Assoc. (1980) 243:549–50. 10.1001/jama.243.6.549 7351788

[97] Van't Hoff W Hoffenberg R London DR Hall R Joplin GF Besser GM Thyroid extract. Br Med J. (1978) 2:200 10.1136/bmj.2.6131.200-c

[98] Smith SR. Desiccated thyroid preparations. obsolete therapy. Arch Int Med. (1984) 144:926–7. 10.1001/archinte.144.5.926 6712407

[99] Jackson IM Cobb WE. Why does anyone still use desiccated thyroid USP? Am J Med. (1978) 64:284–8. 10.1016/0002-9343(78)90057-8 629277

[100] Mayberry WE Gharib H Bilstad JM Sizemore GW. Radioimmunoassay for human thyrotrophin. clinical value in patients with normal and abnormal thyroid function. Ann Intern Med. (1971) 74:471–80. 10.7326/0003-4819-74-4-471 5108040

[101] Hershman JM Pittman JA Jr. Utility of the radioimmunoassay of serum thyrotrophin in man. Ann Intern Med. (1971) 74:481–90. 10.7326/0003-4819-74-4-481 4994544

[102] Hershman JM Clinical application of thyrotropin-releasing hormone. N Engl J Med. (1974) 290:886–90. 10.1056/NEJM197404182901606

[103] Cotton GE Gorman CA Mayberry WE. Suppression of thyrotropin (h-TSH) in serums of patients with myxedema of varying etiology treated with thyroid hormones. N Engl J Med. (1971) 285:529–33. 10.1056/NEJM197109022851001 5109216

[104] Snyder PJ Utiger RD. Inhibition of thyrotropin response to thyrotropin-releasing hormone by small quantities of thyroid hormones. J Clin Invest. (1972) 51:2077–84. 10.1172/JCI107014 4626582 PMC292364

[105] Stock JM Surks MI Oppenheimer JH. Replacement dosage of L-thyroxine in hypothyroidism. a re-evaluation. N Engl J Med. (1974) 290:529–33. 10.1056/NEJM197403072901001 4811096

[106] Evered D Young ET Ormston BJ Menzies R Smith PA Hall R. Treatment of hypothyroidism: a reappraisal of thyroxine therapy. Br Med J. (1973) 3:131–4. 10.1136/bmj.3.5872.131 4720761 PMC1586331

[107] Surks MI Schadlow AR Oppenheimer JH. A new radioimmunoassay for plasma L-triiodothyronine: measurements in thyroid disease and in patients maintained on hormonal replacement. J Clin Invest. (1972) 51:3104–13. 10.1172/JCI107137 4539287 PMC332992

[108] Lieblich J Utiger RD. Triiodothyronine radioimmunoassay. J Clin Invest. (1972) 51:157–66. 10.1172/JCI106786 5007046 PMC332941

[109] Larsen PR Dockalova J Sipula D Wu FM. Immunoassay of thyroxine in unextracted human serum. J Clin Endocrinol Metab. (1973) 37:177–82. 10.1210/jcem-37-2-177 4198255

[110] Surks MI Schadlow AR Stock JM Oppenheimer JH. Determination of iodothyronine absorption and conversion of L-thyroxine (T 4) to L-triiodothyronine (T 3) using turnover rate techniques. J Clin Invest. (1973) 52:805–11. 10.1172/JCI107244 4693647 PMC302327

[111] Inada M Kasagi K Kurata S Kazama Y Takayama H Torizuka K. Estimation of thyroxine and triiodothyronine distribution and of the conversion rate of thyroxine to triiodothyronine in man. J Clin Invest. (1975) 55:1337–48. 10.1172/JCI108053 1133178 PMC301889

[112] Woeber KA Braverman LE The thyroid. In: Ingbar SH editor. Contemporary Endocrinology. New York, NY: Plenum Publishing Corporation (1979). p. 77–117.

[113] Gorman CA Jiang NS Ellefson RD Elveback LR. Comparative effectiveness of dextrothyroxine and levothyroxine in correcting hypothyroidism and lowering blood lipid levels in hypothyroid patients. J Clin Endocrinol Metab. (1979) 49:1–7. 10.1210/jcem-49-1-1 447807

[114] Braverman LE Vagenakis A Downs P Foster AE Sterling K Ingbar SH. Effects of replacement doses of sodium L-thyroxine on the peripheral metabolism of thyroxine and triiodothyronine in man. J Clin Invest. (1973) 52:1010–7. 10.1172/JCI107265 4700481 PMC302354

[115] Samuels MH Kolobova I Smeraglio A Peters D Purnell JQ Schuff KG Effects of levothyroxine replacement or suppressive therapy on energy expenditure and body composition. Thyroid. (2016) 26:347–55. 10.1089/thy.2015.0345 26700485 PMC4790206

[116] Samuels MH Kolobova I Antosik M Niederhausen M Purnell JQ Schuff KG. Thyroid function variation in the normal range, energy expenditure, and body composition in L-T4-treated subjects. J Clin Endocrinol Metab. (2017) 102:2533–42. 10.1210/jc.2017-00224 28460140 PMC5505196

[117] Duntas LH. Thyroid disease and lipids. Thyroid. (2002) 12:287–93. 10.1089/10507250252949405 12034052

[118] Franklyn JA Daykin J Betteridge J Hughes EA Holder R Jones SR. Thyroxine replacement therapy and circulating lipid concentrations. Clin Endocrinol. (1993) 38:453–9. 10.1111/j.1365-2265.1993.tb00339.x 8330440

[119] Hoffenberg R Primary hypothyroidism. In: Ingbar SH Braverman LE editors. Werner's The Thyroid: A Fundamental and Clinical Text. Philadelphia, PA: Lippincott Company JB (1978). p. 1255–66.

[120] Werner SC Treatment. In: Werner SC Ingbar SH editors. The Thyroid a Fundamental and Clinical Text. New York, NY: Harper & Row Publishers, Inc (1978). p. 965–70.

[121] Carr K Mcleod DT Parry G Thornes HM Fine adjustment of thyroxine replacement dosage: comparison of the thyrotrophin releasing hormone tests using a sensitive thyrotrophin assay with measurement of free thyroid hormones and clinical assessment. Clin Endocrinol. (1988) 28:325–33. 10.1111/j.1365-2265.1988.tb01219.x 3139338

[122] Zulewski H Muller B Exer P Miserez AR Staub JJ. Estimation of tissue hypothyroidism by a new clinical score: evaluation of patients with various grades of hypothyroidism and controls. J Clin Endocrinol Metab. (1997) 82:771–6. 10.1210/jc.82.3.771 9062480

[123] Dew R King K Okosieme OE Pearce S Donovan G Taylor P Leese G. Patients' attitudes and perceptions towards treatment of hypothyroidism in general practice: an in-depth qualitative interview study. BJGP Open. (2017) 1:bjgpopen17X100977. 10.3399/bjgpopen17X100977 30564669 PMC6169953

[124] Taylor PN Iqbal A Minassian C Sayers A Draman MS Greenwood R. Falling threshold for treatment of borderline elevated thyrotropin levels-balancing benefits and risks: evidence from a large community-based study. JAMA Int Med. (2014) 174:32–9. 10.1001/jamainternmed.2013.11312 24100714

[125] Medici BB Nygaard B la Cour JL Grand MK Siersma V Nicolaisdottir DR. Changes in prescription routines for treating hypothyroidism between 2001 and 2015: an observational study of 929,684 primary care patients in copenhagen. Thyroid. (2019). 10.1089/thy.2018.0539. [Epub ahead of print]. 31017048

[126] Feller M Snel M Moutzouri E Bauer DC de Montmollin M Aujesky D. Association of thyroid hormone therapy with quality of life and thyroid-related symptoms in patients with subclinical hypothyroidism: a systematic review and meta-analysis. JAMA. (2018) 320:1349–59. 10.1001/jama.2018.13770 30285179 PMC6233842

[127] Medici BB la Cour JL Michaelsson LF Faber JO Nygaard B. Neither baseline nor changes in serum triiodothyronine during levothyroxine/liothyronine combination therapy predict a positive response to this treatment modality in hypothyroid patients with persistent symptoms. Euro Thyroid J. (2017) 6:89–93. 10.1159/000454878 28589090 PMC5422753

[128] Nygaard B Jensen EW Kvetny J Jarlov A Faber J. Effect of combination therapy with thyroxine (T4) and 3,5,3'-triiodothyronine versus T4 monotherapy in patients with hypothyroidism, a double-blind, randomised cross-over study. Euro J Endocrinol. (2009) 161:895–902. 10.1530/EJE-09-0542 19666698

[129] Escobar-Morreale HF Botella-Carretero JI Gomez-Bueno M Galan JM Barrios V Sancho J. Thyroid hormone replacement therapy in primary hypothyroidism: a randomized trial comparing L-thyroxine plus liothyronine with L-thyroxine alone. Ann Intern Med. (2005) 142:412–24. 10.7326/0003-4819-142-6-200503150-00007 15767619

[130] Leese GP Soto-Pedre E Donnelly LA Liothyronine use in a 17 year observational population-based study - the tears study. Clin Endocrinol. (2016) 85:918–25. 10.1111/cen.13052 26940864

[131] Garber JR Cobin RH Gharib H Hennessey JV Klein I Mechanick JI. Clinical practice guidelines for hypothyroidism in adults: cosponsored by the American association of clinical endocrinologists and the american thyroid association. Thyroid. (2012) 22:1200–35. 10.1089/thy.2012.0205 22954017

[132] Torlontano M Durante C Torrente I Crocetti U Augello G Ronga G. Type 2 deiodinase polymorphism (threonine 92 alanine) predicts L-thyroxine dose to achieve target thyrotropin levels in thyroidectomized patients. J Clin Endocrinol Metab. (2008) 93:910–3. 10.1210/jc.2007-1067 18073314

[133] Jonklaas J Davidson B Bhagat S Soldin SJ. Triiodothyronine levels in athyreotic individuals during levothyroxine therapy. JAMA. (2008) 299:769–77. 10.1001/jama.299.7.769 18285588

[134] Woeber KA. Levothyroxine therapy and serum free thyroxine and free triiodothyronine concentrations. J Endocrinol Invest. (2002) 25:106–9. 10.1007/BF03343972 11929079

[135] Alevizaki M Mantzou E Cimponeriu AT Alevizaki CC Koutras DA TSH may not be a good marker for adequate thyroid hormone replacement therapy. Wiener Klinische Wochenschrift. (2005) 117:636–40. 10.1007/s00508-005-0421-0 16416346

[136] Jonklaas J Tefera E Shara N. Short-term time trends in prescribing therapy for hypothyroidism: results of a survey of american thyroid association members. Front Endocrinol. (2019) 10:31. 10.3389/fendo.2019.00031 30761091 PMC6363658

[137] Sawin CT Surks MI London M Ranganathan C Larsen PR. Oral thyroxine: variation in biologic action and tablet content. Ann Intern Med. (1984) 100:641–5. 10.7326/0003-4819-100-5-641 6546843

[138] Jonklaas J Razvi S Reference intervals in the diagnosis of thyroid dysfunction: treating patients not numbers. Lancet Diabetes Endocrinol. (2019) 7:473–83. 10.1016/S2213-8587(18)30371-1 30797750

[139] Dora JM Machado WE Rheinheimer J Crispim D Maia AL. Association of the type 2 deiodinase Thr92Ala polymorphism with type 2 diabetes: case-control study and meta-analysis. Euro J Endocrinol. (2010) 163:427–34. 10.1530/EJE-10-0419 20566590

[140] Wouters HJ van Loon HC van der Klauw MM Elderson MF Slagter SN Kobold AM. No effect of the Thr92Ala polymorphism of deiodinase-2 on thyroid hormone parameters, health-related quality of life, and cognitive functioning in a large population-based cohort study. Thyroid. (2017) 27:147–55. 10.1089/thy.2016.0199 27786042

[141] Cho YY Kim HJ Jang HW Kim TH Ki CS Kim SW. The relationship of 19 functional polymorphisms in iodothyronine deiodinase and psychological well-being in hypothyroid patients. Endocrine. (2017) 57:115–24. 10.1007/s12020-017-1307-4 28466400

[142] Canani LH Capp C Dora JM Meyer EL Wagner MS Harney JW. The type 2 deiodinase A/G (Thr92Ala) polymorphism is associated with decreased enzyme velocity and increased insulin resistance in patients with type 2 diabetes mellitus. J Clin Endocrinol Metab. (2005) 90:3472–8. 10.1210/jc.2004-1977 15797963

[143] Peeters RP van Toor H Klootwijk W de Rijke YB Kuiper GG Uitterlinden AG. Polymorphisms in thyroid hormone pathway genes are associated with plasma TSH and iodothyronine levels in healthy subjects. J Clin Endocrinol Metab. (2003) 88:2880–8. 10.1210/jc.2002-021592 12788902

[144] Castagna MG Dentice M Cantara S Ambrosio R Maino F Porcelli T. DIO2 Thr92Ala reduces deiodinase-2 activity and serum-T3 levels in thyroid-deficient patients. J Clin Endocrinol Metab. (2017) 102:1623–30. 10.1210/jc.2016-2587 28324063

[145] Da Conceicao RR Fernandes GW Fonseca TL Bocco B Bianco AC. Metal coordinated poly-zinc-liothyronine provides stable circulating triiodothyronine levels in hypothyroid rats. Thyroid. (2018) 28:1425–33. 10.1089/thy.2018.0205 30301431 PMC7207055

